# Gliosarcoma with direct involvement of the oculomotor nerve: Case report and literature review

**DOI:** 10.1016/j.radcr.2022.01.018

**Published:** 2022-02-04

**Authors:** Sergio Corvino, Carmela Peca, Giuseppe Corazzelli, Francesco Maiuri

**Affiliations:** Department of Neurosciences and Reproductive and Odontostomatological Sciences, Neurosurgical Clinic; University “Federico II” of Naples, “Federico II”, 5, Via S. Pansini, Naples 80131, Italy

**Keywords:** Gliosarcoma, Oculomotor nerve, Third cranial nerve

## Abstract

Gliosarcoma is a rare malignant brain tumor, characterized by a biphasic tissue pattern with alternating areas displaying glial and mesenchymal differentiation. We first report a case of temporo-mesial gliosarcoma, extended to the crural and ambient cisterns, with direct involvement of the ipsilateral third cranial nerve and encasement of anterior choroidal, posterior communicant and posterior cerebral arteries, presenting without symptoms of peripheral neuropathy. A 61-year-old woman with 1-month history of intense bilateral frontal-temporal headache resistant to pharmacological therapy and paresis of the left lower midface underwent surgical resection, through pterional trans-sylvian approach, of a right temporo-mesial gliosarcoma which directly involved the ipsilateral oculomotor nerve. Reported cases of gliomas with direct involvement of a cranial nerve, from the third to the twelfth, are very rare, whit no cases of gliosarcoma described. Because of its rarity, sometimes this entity is not considered as diagnostic hypothesis and is misdiagnosed, both during preoperative diagnostic evaluation and during the surgery. Gliosarcoma is a strong challenge for neurosurgeons and neurooncologists because of low incidence, poor prognosis and limited reported cases on literature. This case shows unique features for localization, pattern of growth and clinical presentation.

## Introduction

Gliosarcoma, a variant of IDH-wildtype glioblastoma, is a rare malignant brain tumor (incidence 2%-8% of all glioblastoma), characterized by a biphasic tissue pattern with alternating areas displaying glial and mesenchymal differentiation [1]. It displays slight prevalence for sex male and the mean age of affected patients ranges from fifth to seventh decade [1,2]. It more frequently affects the cerebral hemispheres, with a predilection for the temporal lobes [2], [3], [4], [5], [6], [7], [8], [9], followed by the rarer localizations at the posterior cranial fossa and spinal cord. The direct involvement of a cranial nerve by gliomas is very rare [10,11], and no cases of gliosarcoma are described. We first report a case of temporo-mesial gliosarcoma, with extension to the crural and ambient cisterns, with direct involvement of the ipsilateral third cranial nerve and encasement of the anterior choroidal, posterior communicant and posterior cerebral arteries, presenting without symptoms of peripheral neuropathy.

## Case report

**Fig. 1:**
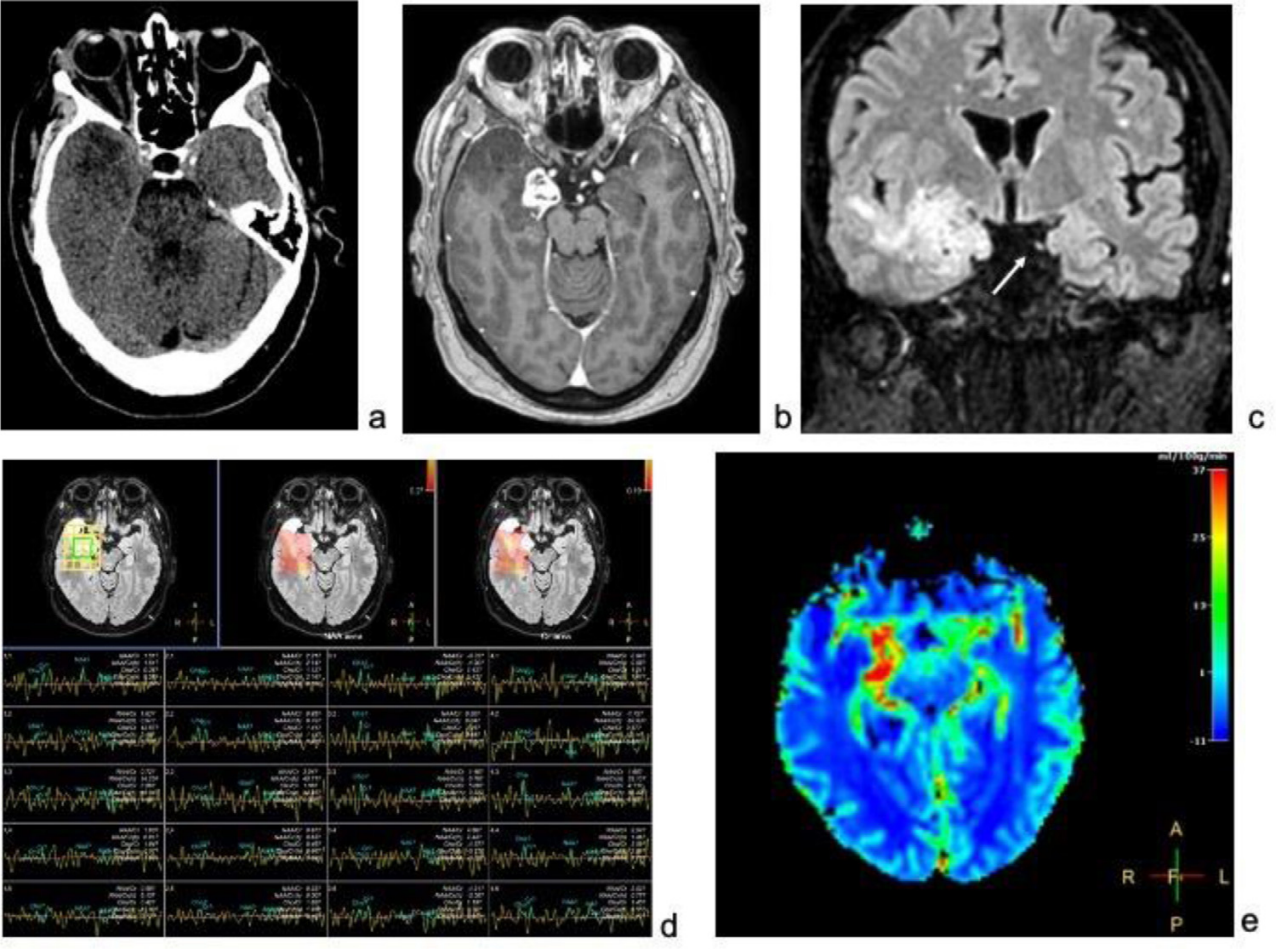
(A) Brain computed tomography showing a right temporal cortico-subcortical area of heterogeneous and slight hypodensity; (B) Brain post contrast MRI axial T1 sequence, demonstring a right temporal infiltrating mass, with intense and heterogeneous enhancement after contrast, irregularly shaped extended from the temporal pole anteriorly to the atrium of the lateral ventricle posteriorly and, through the para-hippocampal gyrus and uncus, to the ambiens and parasellar cisterns medially, with involvement of the homolateral third cranial nerve in its intracisternal tract and encasement of anterior choroidal, posterior communicant and posterior cerebral arteries; (C) Brain MRI, coronal FLAIR sequence, showing the complete sleeve involvement of the right oculomotor nerve compared to the contralateral which instead is well identifiable (white arrow); (D) Brain MRI spectroscopy sequence showing, at lesion level, N-Acetylaspartate (NAA) decreased and Choline (Cho) increased; (E) Brain MRI perfusion showing significant increment of relative Cerebral Blood Volume (rCBV) values at the lesion.

A 61-year-old woman with 1-month history of intense bilateral frontal-temporal headache resistant to pharmacological therapy and paresis of the left lower midface was observed. The brain computed tomography (Fig. 1A) showed a right temporal cortico-subcortical area of heterogeneous and slight hypodensity; a brain MRI detected a right temporal infiltrating mass, hypointense in T1, hyperintense in T2, with intense and heterogeneous enhancement after contrast administration, with irregular shape and extended from the temporal pole, anteriorly, to the atrium of the lateral ventricle, posteriorly, and through the para-hippocampal gyrus and uncus, to the ambiens and parasellar cisterns medially, with involvement of the homolateral third cranial nerve in its intracisternal tract and encasement of the anterior choroidal, posterior communicant and posterior cerebral arteries (Figs. 1B and C). The imaging diagnostic exams were integrated by spectroscopy and perfusion sequences which showed, at the lesion level, decreased N-Acetylaspartate (NAA) and increased Choline (Cho) values, respectively, and significant increment of relative Cerebral Blood Volume (rCBV) values, (Figs. 1D and E). These data were in the first instance consistence with the hypothesis of high-grade glial cell tumor.

The neurologic examination at admission showed static signs of central paresis of the seventh cranial nerve on the left side while no deficit of the third cranial nerve involved by the lesion was evident after accurate clinical exploration.

The patient underwent right pterional trans-sylvian approach. Firstly, the surgical removal was addressed to the tumoral component involving the temporal pole, which appeared as a soft, greyish-pink mass, with central necrotic yellowish area, moderately vascularized; then, once sylvian fissure was opened, the neoplastic component involving the medial part of the temporal lobe and extending to the ambiens and crural cisterns was exposed: it appeared as a firm greyish mass with a small yellowish central core. This last component was strongly adherent to the cisternal part of third cranial nerve and encompassed the anterior choroidal, the posterior communicant and the posterior cerebral arteries, thus the complete resection was not possible.

Postoperative course was characterized by transient right ptosis and mydriasis, which disappeared on the POD 3, and by improvement of the central paresis signs of contralateral midface. Post-contrast brain MRI performed 48 hours after surgical procedure showed a satisfactory tumor removal, with a small residual area of contrast enhancement.

**Fig. 2:**
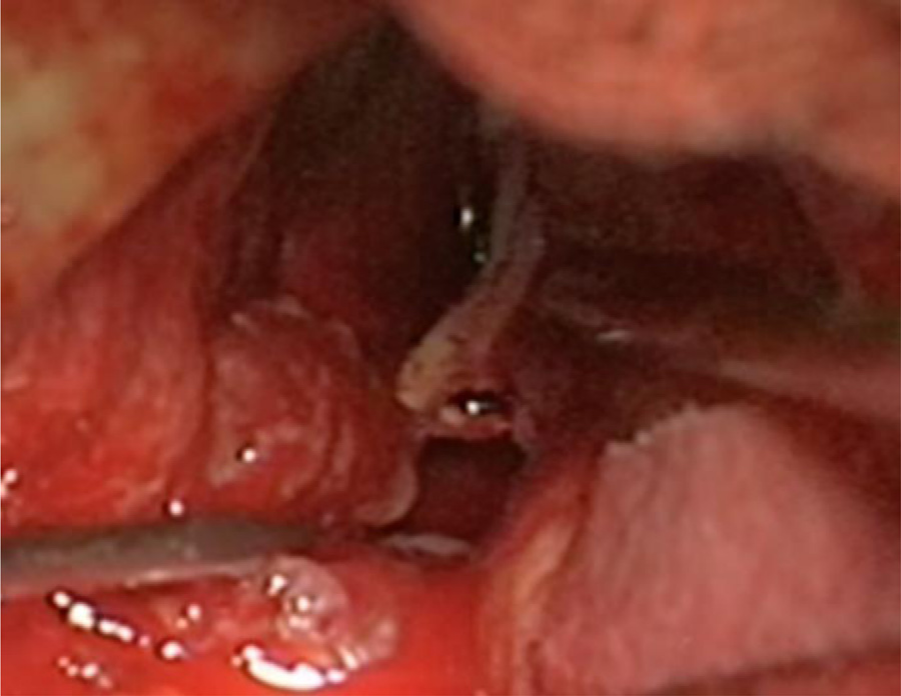
Intraoperative view of right III cranial nerve with proximal tract involved by the lesion and distal free.

**Fig. 3:**
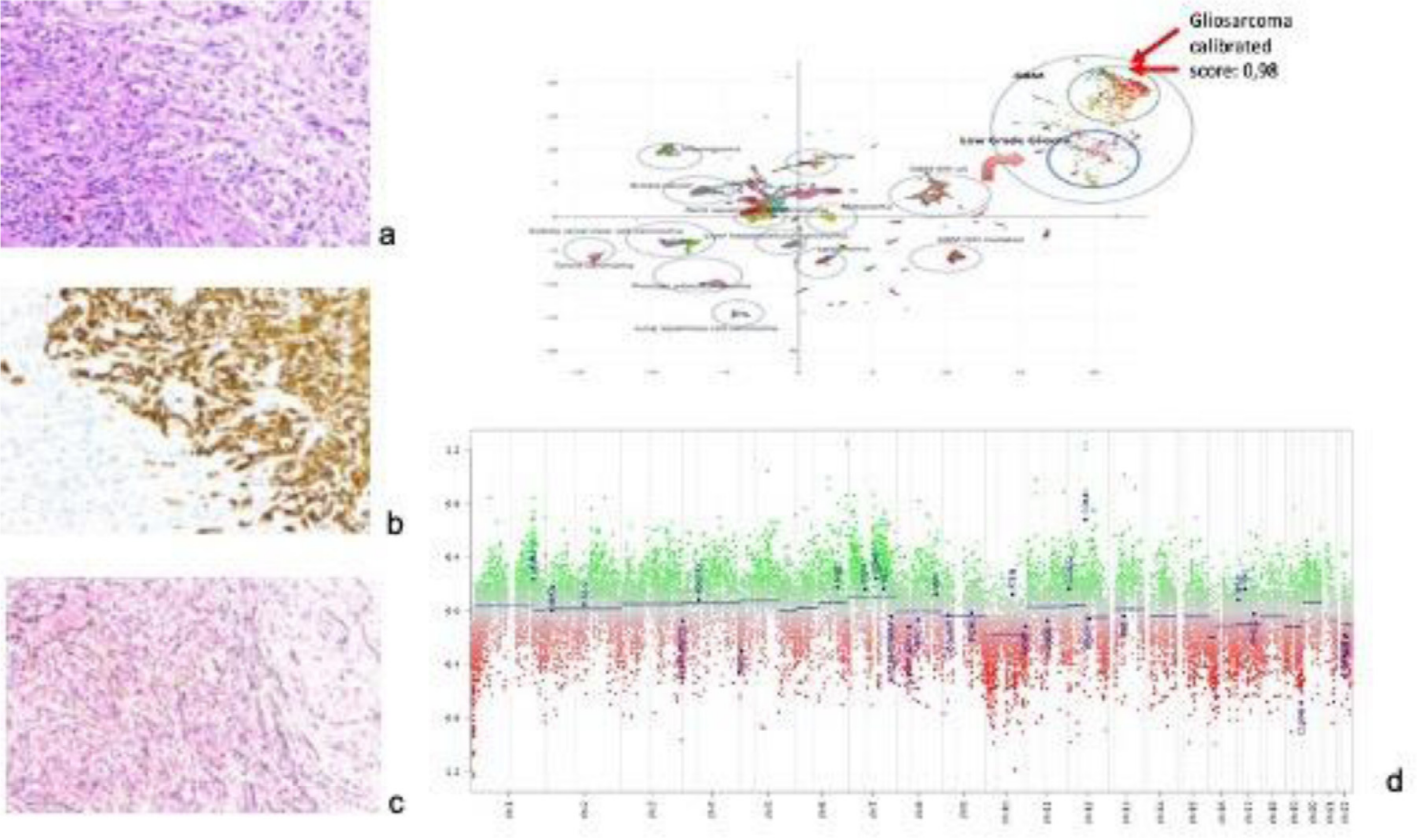
(A) Immunohistochemical exam reporting a biphasic tissue pattern with alternating areas displaying glial and mesenchymal differentiation; (B) the immunophenotype showing GFAP positive, in glial, and negative, in mesenchymal component; (C) reticulin positivity; (D) genetic profile showing lack of mutations in IDH1 and IDH2 genes, no 1p/19q co-deletion, no MGMT methylation, EGFR amplification, gain of 7 chromosome and loss of 10.

The histological and immunohistochemical studies (Fig. 2A) reported a biphasic tissue pattern with alternating areas displaying glial and mesenchymal differentiation; the immunophenotypic characterization revealed GFAP positive expression in glial, and negative in mesenchymal component, whereas p53 was positive in both; all these findings were consistent for the diagnosis of gliosarcoma; genetic profile (Fig. 2B) showed lack of mutations in IDH1 and IDH2 genes, absence of 1p/19q co-deletion, absence of MGMT methylation, EGFR amplification, gain of 7 chromosome and loss of 10, confirming the definitive diagnosis of mesenchymal glioblastoma (Fig. 3).

At discharge, the patient was addressed to the oncological team for the Stupp protocol administration.

No progression-disease was detected on brain MRI with contrast performed 3 months after surgery.

## Discussion

Reported cases of gliomas with direct involvement of a cranial nerve, from the third to the twelfth, were very rare [10], [11], [12], [13], [14], [15], [16], [17], [18], [19], [20], [21], [22], [23], [24] (Table 1); among them the glioblastoma (WHO grade IV) was the most common (11 cases [10,11,14,[22], [23], [24]]), followed by the low-grade gliomas; no cases of gliosarcoma were described. The anatomical region and the cranial nerves more often affected were the cerebellopontine angle and trigeminal and vestibulocochlear nerves, while the oculomotor nerve was involved only 2 times. In most of the cases clinical symptoms related to the cranial nerve directly involved were present; only 5 cases of high-grade temporal glioma associated to isolated third cranial nerve palsy were reported in the literature [11,[25], [26], [27], [28]]. We first report a case of temporo-mesial gliosarcoma, with exophytic pattern of growth to the ambient and crural cisterns and with sleeve involvement of the ipsilateral third cranial nerve, with encasement of the anterior choroidal, posterior communicant and posterior cerebral arteries, presenting without symptoms of peripheral neuropathy.

**Table 1:** Literature review of gliomas with direct cranial nerves involvement.

Authors	Age/sex	Glioma type - WHO grade	Origin	CN involved and side	Neuropathy
Cushing et al. 1917 [12]	n.a.	n.a.	CPA	VIII	Y
Panse et al. 1904 [13]	n.a.	n.a.	n.a.	VIII	n.a.
Wu et al. 2011 [14]	60, M	GBM - IV	CPA	VIII	Y
Mirone et al. 2009 [15]	12, M	Pilocytic Astrocytoma	CPA	VIII	Y
Ree et al. 2005 [16]	36, F	Astrocytoma	Brainstem	V-VII-VIII	Y
Arnautovic et al. 2000 [17]	9, F	Pilocytic Astrocytoma	CPA	V	Y
Takada et al. 1999 [18]	8, F	Pilocytic Astrocytoma	CPA	VII-VIII	Y
Beutler et al. 1995 [19]	58, M	Pilocytic Astrocytoma	CPA	VIII	Y
Forton et al. 1992 [20]	35, F	Astrocytoma	Cerebellum		Y
Kasantikul et al. 1980 [21]	33, F	Astrocytoma	CPA	VIII R	Y
Mabray et al. 2017 [10]	67, M	GBM - IV	Pons	V R	Y
Mabray et al. 2017 [10]	53, F	GBM - IV	Pons, frontal	VIII R	N
Mabray et al. 2017 [10]	67, F	Diffuse Astrocytoma - II	Pons	V L	Y
Mabray et al. 2017 [10]	49, F	GBM - IV	Midbrain, frontal	III R	Y
Mabray et al. 2017 [10]	22, M	GBM - IV	Pons, thalamus, frontal	V R	N
Mabray et al. 2017 [10]	9, M	GBM - IV	Pons, thalamus, midbrain	V R	N
Mabray et al. 2017 [10]	34, M	Oligodendroglioma II	Pons, parietal	V R	N
Mabray et al. 2017 [10]	24, F	GBM - IV	Pons	V-VII R	Y
Breshears et al. 2015 [22]	67, M	GBM – IV	TREZ	V R	Y
Yang et al. 2019 [23]	55, M	GBM – IV	CPA	VIII R	Y
Takami et al. 2018 [24]	55, m	GBM – IV	CPA	VIII R	Y
Marchesini et al. 2020 [11]	69, M	GBM - IV	Frontal/ Temporal	III L	Y
Present case	61, F	Gliosarcoma - IV	Temporal	III R	N

WHO, World Health Organization; CN, Cranial nerve; F, Female; M, Male; n.a., not available; CPA, Cerebellopontine Angle; TREZ, Trigeminal Root Entry Zone; r, right; l, left; Y, Yes; N, Not.

Because of the small number of cases reported in the literature due to the low incidence of GBM with direct cranial nerve involvement and no cases of gliosarcoma, sometimes this kind of lesion is not considered and is misdiagnosed, both during preoperative diagnostic evaluation and during the surgery, hypothesizing the tumor origin from the nerve with secondary extension to the near brain parenchyma, or from heterotopic neuroglial cell dissemination from leptomeningeal gliomas or gliomatosis [29], or from primary brainstem GBM with extension along adjacent cranial nerve.

Concerning the macroscopic features, based on sarcomatous component amount, gliosarcoma with high connective tissue percentage have the appearance of a firm, well-circumscribed mass, which can be mistaken for a metastasis or, when attached to the dura, for a meningioma, whereas lesions less rich in connective tissue may have features more similar to a glioblastoma. Concerning imaging features, gliosarcoma usually presents as a well-demarcated solid mass, often on peripheral location, with heterogeneous enhancement, moderate or marked surrounding edema and abutting dura [2,6,7,[30], [31], [32], [33], [34]], but without dural attachment or invasion.

The pattern of growth shows a rate of extracranial metastases upon 11% for gliosarcoma [35], while it is under 2% for conventional glioblastoma [36,37].

Glioblastoma and gliosarcoma are similar in terms of clinical behavior, treatment and prognosis [2], while they differ for some features which are unique of gliosarcoma, including major propensity to extracranial metastases, intraoperative findings similar to aggressive meningioma, predilection for temporal lobe location and infrequency EGFR mutation [2]. For the diagnosis, immunohistochemical and molecular studies are crucial.

## Conclusion

Gliosarcoma is a strong challenge for neurosurgeons and neurooncologists because of its low incidence, poor prognosis and limited reported cases on literature. This case shows unique features for localization, pattern of growth and clinical presentation.

## Patient consent

Informed consent was obtained from the patient involved in this case.
